# Beyond prevention: Regulating responses to self‐regulation failure to avoid a set‐back effect

**DOI:** 10.1111/aphw.12302

**Published:** 2021-08-18

**Authors:** Marieke A. Adriaanse, Pam ten Broeke

**Affiliations:** ^1^ Department of Social, Health and Organizational Psychology Utrecht University Utrecht The Netherlands; ^2^ Behavioural Science Institute Radboud University Nijmegen The Netherlands

**Keywords:** attributions, failure, self‐efficacy, self‐regulation, setback effect

## Abstract

Despite having good intentions, people fail at times to self‐regulate. Most of these instances of everyday self‐regulation failure are in themselves trivial. However, the ensuing chain of attributions, thoughts, and subsequent behaviors that people experience after an instance of failure may be detrimental to their long‐term self‐regulation success. In two studies, we examined the potential of intervening in the aftermath of failure to prevent this so‐called “setback effect” by instructing people that setbacks may occur and to attribute future incidents of failure to external causes. In Study 1, we tested whether the intervention indeed decreased the frequency of self‐regulation failure in the context of dieting and procrastination. In Study 2, we aimed to replicate the findings from Study 1 in the context of procrastination, and we explored the mediating role of self‐efficacy. In both studies, participants in the intervention condition experienced less self‐regulation failure and more subjective self‐regulation success in the days after the intervention. Study 2 demonstrated that this effect was partly mediated by an increase in self‐efficacy. Taken together, findings suggest that a simple mindset manipulation promoting external attributions to failure may be effective in preventing a setback effect from occurring by protecting self‐efficacy.

## INTRODUCTION

People experience minor failures in self‐regulation on a daily basis, such as raising ones voice while intending to be calm or eating a piece of cake despite being on a diet. To illustrate, when we presented a sample of 100 adults a list of 12 goals (e.g., saving money and eating healthily), they indicated that they actively pursued on average five of these and to have experienced failure on approximately half of these goals in the past 24 h alone (Adriaanse & Ten Broeke, in preparation). Indeed, despite the emerging different perspectives about the nature or mechanisms of self‐control (e.g., Milyavskaya & Inzlicht, 2018), research on self‐control generally shows that people are simply not equipped to regulate all of their behavior all of the time (De Ridder et al., 2018). In addition to these inherent limits in self‐control, people typically pursue a multitude of goals simultaneously, which will at times conflict, such as when a goal to study for exams conflicts with a goal to exercise. These conflicting goals inevitably result in experiencing instances of failure regardless of the ability, capacity, or motivation to self‐regulate.

Fortunately, in reality, many instances of failure are in themselves rather trivial (Baumeister & Heatherton, 1996). For example, on their own, violations such as skipping one night of your exercise regime or eating one piece of cake hardly pose a serious threat to your long‐term goal to lose 5 pounds in the next 3 months. Yet, a single incident of failure may nonetheless affect long‐term self‐regulation success when people make maladaptive causal attributions to explain their behavior. In fact, building on research on the abstinence violation effect (AVE) in the addiction literature (Marlatt & George, 1984), these so‐called “lapse activated patterns” resulting from maladaptive attributions for failure were listed as “one of the seven deathly threats to self‐regulation” (Wagner & Heatherton, 2015). Recently, Wenzel et al. (2020) demonstrated that also in the context of everyday self‐regulation people are indeed more likely to fail after experiencing an initial instance of failure. Wenzel et al. dubbed this effect “the setback effect,” and we will adopt this terminology for the remainder of this paper.

Based on their findings, Wenzel et al. (2020) highlighted the importance of preparing individuals for setbacks in self‐regulation interventions. However, a typical self‐regulation intervention teaches participants various strategies and skills to *prevent* self‐regulation failure, such as goal setting (e.g., Swoboda et al., 2017), planning (e.g., Luszczynska & Schwarzer, 2003), and progress monitoring (e.g., Steinberg et al., 2013). We know of few interventions that teach participants that experiencing some instances of failure will simply be inevitable in practical terms, let alone how to adequately respond to such setbacks. As such, the aim of the present research is to assess whether and how a simple intervention addressing individuals' cognitive responses in the aftermath of failure can prevent a setback effect from occurring.

Based on attribution theory and the AVE, the intervention tested in this research targets people's causal attributions regarding their self‐regulation failure. According to attribution theory (Heider, 1958), people have a strong tendency to understand their behavior and to determine its causes. While some instances of self‐regulation failure can be straightforwardly explained, such as when illness prevents you from exercising, often the reasons for self‐regulation failure are ambiguous or even inaccessible (Sheeran et al., 2013). For example, a dieter might indulge in a high caloric snack as a result of context cues of which they are unaware or which they underestimate, such as TV food advertisements (Harris et al., 2009) or other people's eating behavior (Tanner et al., 2008). In these situations, even though people lack insights into the processes triggering their behavior, they still experience a need to explain their behavior as a result of experiencing an inconsistency between their behavior and their goals (Oettingen et al., 2006; Parks‐Stamm et al., 2010). As a consequence, people often make erroneous attributions regarding the causes of their goal violations (Adriaanse et al., 2014; Bar‐Anan et al., 2010; Nisbett & Wilson, 1977).

These attributions occurring in the aftermath of failure, more so than the incident of failure in itself, may be particularly destructive to long‐term self‐regulation success depending on the locus of causality. That is, according to the AVE, when addicts experience a single lapse in abstinence behavior (e.g., smoking), and they attribute the lapse to stable, internal factors beyond their control (e.g., “I have no willpower”), they are more likely to fully relapse into the addiction behavior afterwards. In contrast, when addicts attribute a lapse to external circumstances that are specific to the particular lapse and experienced as less threatening, they are less likely to relapse (Curry et al., 1987). In the present research, we build on these findings and tested whether, in the context of everyday self‐regulation, a brief mindset manipulation that promotes external attributions in the aftermath of failure can prevent the setback effect from occurring.

The second aim of the present research was to explore the underlying mechanism by which adopting external attributions might diminish the likelihood of a setback effect. A promising candidate is self‐efficacy. Self‐efficacy can be defined as an individual's belief in their capacity to implement a behavior needed to reach their goal (Bandura, 1978). It is a central predictor in many behavior change theories (e.g., Maddux & Rogers, 1983; Schwarzer, 2008). People derive their self‐efficacy from their performance in previous situations (Bandura, 1978). According to Marlatt and Gordon (1985), when people experience failure and do not have access to plausible external explanations for behavior, they attribute lapses internally and take it as an indicator that they are incapable (i.e., they experience a decrease in self‐efficacy). This, in turn, negatively affects individuals' future attempts to regulate their behavior (Marlatt & Witkiewtiz, 2005). This suggest that the effect of causal attributions on subsequent self‐regulation success is likely mediated by changes in self‐efficacy and that a mindset manipulation that stimulates external attributions could prevent a setback effect through maintaining people's self‐efficacy upon being confronted with failure.

### The present studies

In the present paper, we present two studies designed to test whether an intervention teaching people that setbacks may occur and that they may be attributable to external causes, maintains self‐efficacy and prevents failure from spilling over to subsequent situations. Specifically, in Study 1, we tested whether the intervention indeed prevented a setback effect. In Study 2, we aimed to replicate the findings from Study 1, and we explored the mediating role of self‐efficacy.

Statistical analyses were performed in R (Version 3.6.3). See the Supporting Information for information about handling of assumptions and outliers and for materials of all studies. Unless otherwise indicated, statistical assumptions were met, or the statistical test was expected to be robust against violations. Studies reported in this paper were approved by the Ethics Committee of the Faculty of Social and Behavioural Sciences of Utrecht University.

## STUDY 1

We designed a brief intervention targeting responses to future incidents of failure by instructing participants in the experimental condition to attribute failure externally. The intervention was designed with two criteria in mind. First, it was deemed crucial that the intervention was brief and simple to implement by individuals without therapist involvement. Second, the instructions of the interventions should be flexible so it could apply to various unforeseen future situations in which failure may occur. In Study 1, we tested the effect of this intervention using a randomized controlled design, comparing two groups of participants receiving the intervention with a group of participants receiving no intervention (control group) on how many times they failed and how much self‐regulation success they experienced in the days after the intervention. Behavior change interventions often have spill‐over effects to other behavioral domains that were not originally targeted in the intervention (Dolan & Galizzi, 2015). To test for such a potential spill‐over effect, we included two intervention groups: one received an intervention focused on eating behavior, and one received an intervention focused on procrastination behavior.^1^

### Method

#### Participants

Three hundred twenty‐two female, native English participants, aged 18–30, currently residing in the United Kingdom were recruited through Prolific. We included only females aged 18–30 to create a relatively homogeneous group for whom dieting is a relevant concern (Weiss et al., 2006). The sample size was based on an a priori power analysis in G*Power for an analysis of variance (ANOVA) testing the effect of condition on one of the two dependent variables, yielding a sample size of *N* = 326, aiming for a power of .90 (*α* = .05), an approximately medium effect size of *f* = .22 (assuming a 25% reduction in failure as smallest effect of interest and expected variance based on pilot studies) and accounting for a dropout of 23% (based on pilot studies). We only included participants who were currently restricting their food intake with the goal to manage their weight. Twenty‐four participants dropped out. The final sample consisted of 298 participants with an average age of 24.84 years (SD = 3.59) and an average BMI of 25.11 (SD = 6.37). Regarding highest obtained educational degree, most participants had a high‐school or secondary school degree (41%), followed by a bachelor's degree (40%), a master's degree (13%), an associate degree (6%), a PhD (<1%), or no degree (<1%).

#### Design and procedure

We used a randomized controlled longitudinal design. At T1, participants were randomly assigned to one of three conditions: an intervention in the domain of dieting (dieting intervention; *N* = 98), an intervention in the domain of procrastination (procrastination intervention; *N* = 100), or a control condition (*N* = 100). At T2, we assessed the dependent variables: dieting failure T2, dieting success T2, procrastination failure T2, and procrastination success T2, which were aggregated scores over four subsequent days. For each intervention, we investigated the effect on the outcome measures concerning the same behavioral domain (domain‐congruent effect) as well as the other behavioral domain (domain‐incongruent effect).

After informed consent, demographic information, and general dieting and procrastination behavior was assessed. Next, participants were randomly assigned to one of the conditions. The intervention consisted of two parts: reading an informational text, and formulating an if‐then plan. It took participants approximately 10 min to finish part one, and all data for part one was collected within 1 day. Four days after part one at 7 a.m., participants were invited to complete part two of the study within 24 h (before 7 a.m. the next day). Participants indicated the number of failure instances (failure T2) and their perception of success (success T2) regarding their dieting and procrastination behavior, for the past 4 days.^2^ The study ended with a debriefing and reimbursement of £4.50. It took participants approximately 7 min to finish part two.

#### Materials

##### Baseline dieting and procrastination questions

Baseline dieting and procrastination were assessed with 12 questions: one question on baseline failure and one question on baseline success for each of 3 days (i.e., Friday, Saturday, and Sunday) for each behavioral domain. Participants were instructed to create a vivid mental image of a typical Friday/Saturday/Sunday and to indicate (a) how many times they typically eat something that is not in line with their dieting goal/how many minutes they spend procrastinating on tasks over the course of a typical day and (b) to what extent they typically feel like they successfully follow their diet/refrain from procrastinating on a typical Friday/Saturday/Sunday, on a slider from 0% (*Unsuccessful*) to 100% (*Successful*).

### Experimental manipulation

#### Informational text

Participants in the intervention condition read a short informational text (see Supporting Information). The texts were equivalent except for the specific behavior. Participants read:

Research has shown that whether you can get back on track actually has a lot to do with the way people think about the causes of their [unhealthy eating behavior/procrastination].

Next, they read the reattribution text:

A helpful way for you to think about why you failed your [diet/ procrastinated] is to focus on the factors outside of you, such as the environment, or the people around you that influenced your behavior. These factors have a tremendous, but often underestimated, effect on your [eating behavior/procrastination]. Research has shown that acknowledging these kinds of external factors when [failing your diet/procrastinating] may be a helpful way to get you back on track.

Participants in the control condition did not read a text.

#### Implementation intention

The implementation intention was introduced to link the “mindset shift” described in the information text to any future experiences of failure. It stated:

If I fail [to adhere to my dieting goal/to pursue my goal due to procrastination], then I will reflect on the external factors that contributed to [this failure/my procrastinating behavior], and will continue to pursue my [dieting] goal as usual.

Participants were asked to commit to the plan and to repeat and picture it in mind as vivid as possible for a few times over a period of 2 min (Knäuper et al., 2009). Participants in the control condition did not make an implementation intention.

##### Failure and success at T2

Dieting failure T2 was assessed as the sum of four retrospective daily ratings of failure frequency (“How many times during this day did you eat something that was not in line with your dieting goal?”). Dieting success T2 was assessed as the mean of four retrospective daily ratings of dieting success (“To what extent do you feel like you successfully followed your diet during this day?”) answered on a Likert scale ranging from 1 (*not at all*) to 7 (*very much*). For procrastination failure T2 and procrastination success T2, these questions were adapted to the domain of procrastination, and failure T2 was assessed in minutes.

### Results

As the intervention specifically targeted *responses* to failure, participants responding “zero” times to dieting failure T2 (*N* = 1 in the dieting intervention condition, *N* = 4 in the procrastination intervention condition, and *N* = 4 in the control condition) or “zero” minutes to procrastination failure T2 (*N* = 4 in the dieting intervention condition, *N* = 1 in the procrastination intervention condition, and *N* = 1 in the control condition) were excluded from the analyses on all outcome measures for that respective behavior. One participant in the control condition had missing responses on dieting failure T2 and success T2. Twelve participants (*N* = 6 in the dieting intervention, *N* = 5 in the procrastination condition, and *N* = 1 in the control condition) had missing responses on procrastination failure T2 and success T2. These participants were also excluded from the analyses on all outcome measures in that respective behavioral domain. Both dieting failure T2 and procrastination failure T2 were skewed, so we performed a square root transformed version of these variables to normalize the distribution before running statistical tests. Means and standard deviations are presented for the untransformed variables.

#### Descriptives and intercorrelations

See Table 1 for descriptives and intercorrelations. Multiple one‐way ANOVAs indicated that participants in all three conditions did not differ on age, BMI, baseline dieting failure, baseline dieting success, baseline procrastination failure, and baseline procrastination success, *p*s ≥ .063.

**TABLE 1:** Study 1: Means (M), standard deviations (SD), and intercorrelations

	1	2	3	4	5	6	7	8	9	10
Age (1)										
BMI (2)	**0.19**									
Baseline dieting failure (3)	−0.06	**0.19**								
Baseline dieting success (4)	−0.02	**−0.24**	**−0.59**							
Baseline procrastination failure (5)	−0.11	−0.02	**0.06**	−0.09						
Baseline procrastination success (6)	0.04	−0.11	**−0.32**	**0.42**	**−0.13**					
Dieting failure T2 (7)	−0.10	0.07	**0.40**	**−0.24**	**0.18**	**−0.18**				
Dieting success T2 (8)	0.03	−0.11	**−0.27**	**0.41**	**−0.15**	**0.24**	**−0.61**			
Procrastination failure T2 (9)	−0.08	−0.08	**0.13**	0.00	**0.60**	**−0.27**	**0.17**	−0.03		
Procrastination success T2 (10)	**0.17**	−0.02	**−0.22**	**0.26**	**−0.16**	**0.36**	**−0.28**	**0.35**	**−0.52**	
*M*	24.84	25.11	7.62	51.77	344.4	52.11	5.90	55.80	178.30	65.15
SD	3.59	6.37	5.20	21.98	1638.10	20.08	4.11	22.35	187.7	18.03

*Note*: Correlation coefficients in bold are significant (*p* < .05).

#### Main analyses

##### Dieting behavior

To test the effect of the dieting intervention on dieting behavior (domain‐congruent effect), we performed two independent *t*‐tests comparing the dieting intervention with the control condition on dieting failure T2 and dieting success T2. Results indicated that dieting failure T2 was significantly lower in the dieting intervention (*M* = 5.30 *SD* = 3.77) than in the control condition (*M* = 6.36, *SD* = 3.89), *t*(190) = 2.17, *p* = .032, Cohen's *d* = .31. Dieting success T2 was significantly higher in the dieting intervention (*M* = 59.94, *SD* = 21.15) than in the control condition (*M* = 53.26, *SD* = 22.61), *t*(190) = −2.12, *p* = .036, Cohen's *d* = .31.

To test the effect of the procrastination intervention on dieting behavior (domain‐incongruent effect), we performed two independent *t*‐tests comparing the procrastination intervention with the control condition on dieting failure T2 and dieting success T2. Results indicated no effect of the procrastination intervention on dieting failure T2, *p* = .385, or dieting success T2, *p* = .790.

##### Procrastination behavior

To test the effect of the procrastination intervention on procrastination behavior (domain‐congruent effect), we performed two independent *t*‐tests comparing the procrastination intervention with the control condition on procrastination failure T2 and procrastination success T2. Results indicated that procrastination failure T2 was significantly lower in the procrastination intervention (*M* = 137.80, *SD* = 132.90) than in the control condition (*M* = 230.60, *SD* = 231.00), *t*(190) = 3.71, *p* < .001, Cohen's *d* = .54. Procrastination success T2 was marginally significantly higher in the procrastination intervention (*M* = 66.45, *SD* = 16.73) than in the control condition (*M* = 61.39, *SD* = 20.08), *t*(190) = −1.90, *p* = .060, Cohen's *d* = .27.

To test the effect of the dieting intervention on procrastination behavior (domain‐incongruent effect), we performed two independent t‐tests comparing the dieting intervention with the control condition on procrastination failure T2 and procrastination success T2. Results indicated procrastination failure T2 was significantly lower in the dieting intervention (*M* = 163.50, SD = 171.80) than in the control condition (*M* = 230.60, SD = 231.00), *t*(184) = 2.46, *p* = .015, Cohen's *d* = .36. Procrastination success at T2 was significantly higher after the dieting intervention (*M* = 67.94, SD = 16.35) than in the control condition (*M* = 61.39, SD = 20.08), *t*(184) = −2.42, *p* = .016, Cohen's *d* = .36.

### Discussion

The findings of Study 1 show that participants who were instructed to adopt a situated perspective and make external attributions upon experiencing a setback failed less and experienced more success in the days after the intervention, both when the intervention addressed dieting or procrastination behavior. These findings provide evidence for the potential and feasibility of intervening in the aftermath of a setback. Interestingly, participants who learned to adopt a mind‐set change regarding their dieting failures also experienced more success on their procrastination behavior. A simple intervention aimed at preventing the setback effect in one domain might thus spill‐over to positively affect responses to failure in other behavioral domains. We did not directly assess people's cognitive responses to failure. However, we observed that for both eating and procrastination, the intervention did not significantly affect the duration until participants' *first* instance of failure after the intervention (day 1, day 2, or day 3)^3^. This suggests that, as intended, the effect of the intervention only occurred after participants experienced and could adjust their response to a setback.

## STUDY 2

We conducted Study 2 to replicate the effect of the intervention in Study 1. Study 2 only incorporated procrastination behavior. Based on the findings of Study 1, we hypothesized that participants in the intervention condition would report less procrastination failure and more procrastination success compared with participants in the control condition. We additionally investigated whether the effect of the intervention was mediated by changes in self‐efficacy after the intervention. Moreover, as a manipulation check, we also assessed the extent to which participants indeed reflected on internal versus external causes upon failure in the days after the intervention. Combined, this served as a more stringent test of the manipulation, besides a simple monitoring effect. The study was preregistered at AsPredicted^4^: https://aspredicted.org/blind.php?x=3zx99n.

### Methods

#### Participants

Two hundred forty‐eight native English speaking participants, currently residing in the United Kingdom, who had not participated in our other studies on the setback effect, were recruited through Prolific. Originally, we aimed for 260 participants, but due to technical difficulties, 12 participants who did not meet the inclusion criteria were incorrectly included. This sample size was based on an a priori power analysis in G*Power for an ANOVA testing the effect of condition on the outcome, yielding a sample size of *N* = 260, aiming for a power of .90 (*α* = .05), an approximately medium effect size of *f* = .22 (assuming a 25% reduction of failure as smallest effect of interest and expected variance based on pilot studies) and accounting for a dropout of 15% (based on pilot studies and Study 1). We included only participants who indicated to have procrastinated in the past month. Thirty‐eight participants dropped out, and one participant had missing responses on the outcome and was therefore excluded from the analyses. Participants who dropped out did not significantly differ from participants who continued the study on age, baseline procrastination failure and baseline self‐efficacy. The final sample consisted of 209 participants, with an average age of 32.68 years *(SD* = 12.67). One hundred thirty‐eight participants were female, 69 participants were male, and two participants did not specify a gender. Regarding highest obtained educational degree, most participants had a bachelor's degree (40%), followed by a high‐school or secondary school degree (39%), a master's degree (11%), an associate degree (9%), or a PhD (1%).

#### Design, procedure, and materials

The design, procedure, and materials of Study 2 were similar to Study 1 but only included the procrastination intervention condition (*N* = 100) and the control condition (*N* = 109). At T1, we additionally assessed baseline measures of self‐efficacy and intention. At the start of T2, we assessed self‐efficacy as mediator. In addition, at the end of T2, we assessed the extent to which participants had reflected on external and internal factors upon failure in the past days, which served as a manipulation check. It took participants approximately 15 min to finish part one and to finish part two.

##### Baseline self‐efficacy and intention

Baseline self‐efficacy was assessed with two items (Spearman–Brown reliability *ρ* = .84): “I feel in control over minimizing my procrastination behavior” and “I feel confident in my abilities to minimize my procrastination behavior” (Ajzen, 2002). Baseline intention was assessed with two items (*ρ* = .66): “I intend to minimize my procrastination behavior” and “I plan to minimize my procrastination behavior” (Adriaanse et al., 2010). All items were answered on a Likert scale ranging from 1 (*totally disagree*) to 7 (*totally agree*).

##### Self‐efficacy T2

Self‐efficacy at T2 was assessed with two items using a “compared to before” format (*ρ* = .91): “In comparison to before I completed this study, I feel in control over minimizing my procrastination behavior” and “In comparison to before I completed this study, I feel confident in my abilities to minimize my procrastination behavior” (Adriaanse et al., 2010; Ajzen, 2002). The items were answered on a Likert scale ranging from 1 (*totally disagree*) to 7 (*totally agree*).

##### Internal and external reflection

As a manipulation check, participants were asked “In the past 3 days, whenever you procrastinated, to what extend did you reflect on the *external* factors that contributed to that failure?” and “In the past 3 days, whenever you procrastinated, to what extend did you reflect on the *internal* factors that contributed to that failure?”, answered on a Likert scale ranging from 1 (*not at all*) to 7 (*very much*).

### Results

As the intervention targeted responses to failure, participants responding “zero” minutes to procrastination failure T2 (*N* = 4) were excluded from the analyses.^5^ As in Study 1, procrastination failure T2 was skewed, so we performed a square root transformed version of this variables to normalize the distribution before running statistical tests. Means and standard deviations are presented for the untransformed variable.

#### Descriptives and intercorrelations

See Table 2 for descriptives and intercorrelations. Self‐efficacy at T1 was significantly and negatively associated with procrastination failure T2 (*r* = −.28, *p* < .001). Multiple one‐way ANOVAs indicated that participants in all three conditions did not differ on age, baseline self‐efficacy, baseline intention, and baseline procrastination failure, *p*s ≥ .170.

**TABLE 2:** Study 2: Means (M), standard deviations (SD), and intercorrelations

	1	2	3	4	5	6	7	8
Age (1)								
Baseline procrastination failure (2)	0.03							
Baseline self‐efficacy (3)	−0.01	−0.11						
Baseline intention (4)	−0.03	0.13	**0.21**					
Self‐efficacy T2 (5)	−0.03	<0.001	**0.40**	0.10				
Procrastination failure T2 (6)	−0.07	**0.52**	**−0.18**	0.01	**−0.28**			
Internal reflection (7)	−0.02	−0.01	0.07	**0.18**	**0.19**	0.08		
External reflection (8)	−0.05	−0.03	−0.03	**0.15**	**0.26**	−0.12	**0.47**	
*M*	32.68	266.90	3.88	4.85	4.60	179.00	4.48	4.19
SD	12.67	333.40	1.30	1.21	1.24	183.40	1.70	1.79

*Note*: Correlation coefficients in bold are significant (*p* < .05).

#### Manipulation check

As a manipulation check, we performed two independent *t*‐tests comparing the conditions on internal attributions and external attributions at T2. Participants in the intervention condition reflected more on external factors (*M* = 4.92, *SD* = 1.60) compared with participants in the control condition (*M* = 3.52, *SD* = 1.69), *t*(207) = −6.12, *p* < .001. Reflecting on internal factors was not significantly different between the two groups, *p* = .150.

#### Main analyses

##### Intervention and procrastination failure

To test the effect of the intervention on procrastination behavior, we performed an independent *t*‐test comparing the intervention condition with the control condition on procrastination failure T2. As the assumption of equal variance was violated, a Welsh degrees of freedom modification was applied. Results indicated that procrastination failure T2 was significantly lower in the procrastination intervention (*M* = 123.90, *SD* = 101.10) than in the control condition (*M* = 228.50, *SD* = 223.00), *t*(181.70) = 4.28, *p* < .001, Cohen's *d* = .58.

##### Intervention and self‐efficacy

To test the effect of the intervention on self‐efficacy at T2, we performed an independent *t*‐test comparing the intervention condition to the control condition on self‐efficacy at T2. Relative self‐efficacy was higher in the procrastination intervention (*M* = 4.83, *SD* = 1.14) than in the control condition (*M* = 4.39, *SD* = 1.28), *t*(207) = −2.609, *p* = .010.

##### Mediation analyses

The previous analyses confirmed the effect of the intervention on procrastination failure (total effect), the effect of the intervention on the self‐efficacy (alpha path), and the effect of self‐efficacy on procrastination failure (beta path). To test the final step of the simple mediation model (Baron & Kenny, 1986), we tested the indirect effect of the intervention on procrastination failure T2 through self‐efficacy using bias‐corrected bootstrapped 95% confidence intervals. Self‐efficacy significantly mediated the effect of the intervention on procrastination failure T2 (*B* = −0.49, 95% CI [−1.20; −0.10]).

## DISCUSSION

Study 2 replicated the findings of Study 1. When we instructed participants to adopt a situated perspective upon experiencing a setback on their procrastination goal, they on average procrastinated less in the days after. Moreover, we found tentative evidence that this mindset manipulation helps people limit subsequent failure through a boost in their self‐efficacy. That is, participants who were instructed to adopt a situated perspective on failure reported higher feelings of self‐efficacy in the subsequent days compared with participants in the control condition, which in turn was associated with less procrastination. A simple instruction to focus on external factors might thus be a promising intervention to help people respond to inevitable setbacks in a way that protects self‐efficacy and prevent a single setback from increasing subsequent failure.

## GENERAL DISCUSSION

The setback effect, or the potential of a single setback in self‐regulation in increasing the chances of subsequent setbacks, has been proposed as a key threat to long‐term self‐regulation success (Baumeister & Heatherton, 1996; Wagner & Heatherton, 2015; Wenzel et al., 2020). Research on the AVE and attribution theory suggests that individuals are most likely to experience such a setback effect when they make internal attributions for failure, which results in reduced self‐efficacy. Unfortunately, even though experiencing setbacks can be considered an inevitable experience in the context of everyday self‐regulation, current self‐regulation interventions lack guidance on how to adaptively respond to setbacks to prevent it from having a negative effect on subsequent goal striving. With the present research, we therefore aimed to investigate whether a novel simple intervention targeting attribution processes in the aftermath of failure could potentially be used as an effective strategy to prevent the setback effect from occurring.

Specifically, participants in the experimental conditions of Studies 1 and 2 were encouraged to adopt a situated perspective on self‐regulation failure by encouraging them to acknowledge and focus on external factors when experiencing setbacks in the future. As a result, they on average failed their diets once less, and on average procrastinated 93 to 105 min less over a period of 3 days compared with participants in the control condition. Findings in Study 2 indicated that this effect was partly fueled by an increase in people's self‐efficacy. So, by protecting people's self‐efficacy, a simple intervention promoting external attributions in case of future self‐regulation failure could reduce the chance of a setback effect. Moreover, findings of Study 1 provide some preliminary evidence that the effect of these mindset manipulations may spill over to other behavioral domains as well.

Our results suggest that self‐regulation research needs to adopt a broader approach to the topic of self‐regulation failure. Such an approach should acknowledge that experiencing some setbacks is inevitable and put more emphasis on understanding the processes occurring in the aftermath of setbacks rather than directing all of its efforts at preventing failure. Our findings indicate that, without intervention (i.e., the control condition), participants failed their diets approximately six times and procrastinated approximately 230 min over a time period of 4 days. This emphasizes the importance of preparing individuals for setbacks and developing interventions to prevent people from responding to a setback in a maladaptive manner.

In the present study, we focused on causal attributions and self‐efficacy. However, research on the setback effect is limited and more research is needed to investigate other processes that may play a role in the setback effect and could be targeted in interventions as well. For example, the disinhibition effect (Herman & Mack, 1975) suggests that when rigid dieters violate their dieting goals once, they experience a “what the hell effect”: They catastrophize the initial diet violation and feel that any further attempts at regulating their food intake are useless. As a consequence, they subsequently consume *more* calories, instead of compensating for the single setback by pursuing their diets as usual. However, follow‐up studies on the disinhibition effect did not consistently replicate the effect, and researchers have experience difficulties with pinpointing the proposed cognitive underlying mechanism (Jansen et al., 1988). As such, future studies are needed to explore whether decreases in the perceived usefulness to self‐regulate may or may not be an additional underlying mechanism of the setback effect in the broader context of everyday self‐regulation, and a potential target for interventions.

### Limitations and avenues for future research

Several limitations should be noted. First of all, both studies used self‐report measures of behavior, including self‐constructed items for the outcome measures failure and success that were not tested for validity and reliability. Even though we provided several tools to stimulate accurate recall (e.g., providing a calendar) and participants reported adequate abilities to recall their behavior, such self‐report measures are inherently limited. Second, in both studies, we investigated the effect on total failure and success in the days after the invention. Future studies should investigate the dynamics of self‐regulation behavior over longer time periods and more precisely pinpoint the effect of the intervention by using more advance methods such as ecological momentary assessments (EMA) in which people are prompted to report on their goals, temptations, and responses multiple times a day (e.g., see Wenzel et al., 2020). Third, while we concur with Wenzel et al. (2020) that it is important to prepare individuals for setbacks in self‐regulation interventions, strategies focusing on preventing failure are still necessary and the present manipulation should therefore ideally be used alongside other strategies such as goal setting (e.g., Swoboda et al., 2017), and planning (e.g., Luszczynska & Schwarzer, 2003) in an intervention. Whether or not our mindset manipulation is still effective when embedded in existing interventions remains a question to be explored in future research. Finally, in Study 1, we included only females aged 18 to 30, with the goal to select individuals for whom dieting is a relevant concern (Weiss et al., 2006). Even though the intervention effect on procrastination behavior was replicated using a wider, more representative sample in Study 2, future research should explore the generalizability of the intervention effect on dieting behavior to other populations.

Our finding that the effect of the intervention was mediated by self‐efficacy suggests that besides promoting external attributions to protect self‐efficacy, future interventions could also directly target self‐efficacy. An example of an intervention directly boosting self‐efficacy could be to help people refocus on previous successes, which has been found successful in physical activity (Ashford et al., 2010). Finally, results of Study 1 suggest that there may be a potential for spill‐over effects across domains, which is promising considering the multitude of goals that people pursue on a daily basis. However, this effect needs to be further explored and replicated in further studies before drawing firm conclusions about this possibility.

### Concluding remarks

Experiencing setbacks is inevitable. Although a setback may in itself be harmless, depending on subsequent changes in relevant cognitions, such as people's confidence in their abilities to regulate their behavior, it could be the starting point of a slippery slope toward more failure. With the present research, we provide tentative evidence for the feasibility and effectiveness of intervening in the aftermath of failure to prevent such a set‐back from occurring. In doing so, we address an important gap in the literature on self‐regulation and self‐regulation interventions, which have been primarily focused on preventing failure and which at present lack strategies to help individuals to adequately respond to setbacks.

## Supporting information


**Data S1.** Supporting Information

## Data Availability

All data that we used for our analyses, and R code for data processing, analysis, and visualization, will be made available on the Open Science Framework: https://osf.io/fwqxb/?view_only=b074c24135da4f6e815a9d25f6f94a2f.

## ENDNOTES

1 This study was originally preregistered. However, certain assumptions were adapted through the course of the study, and therefore, we decided to withdraw the preregistration and consider all analyses and findings of this study as exploratory.

2 In both Study 1 and Study 2, participants additionally answered questions on the extent to which they were able to recall their dieting and procrastination behavior of the respective day (see the Supporting Information). Average recall was high in all studies (*M*s ≥ 4.88 on a 7‐point scale) and additional analyses revealed that excluding participants with low average recall (<4) did not change the pattern of results.

3 Chi‐square tests of independence for both eating behavior and procrastination behavior indicated that the association between condition (eat intervention vs. control; or procrastination intervention vs. control) and *time until first failure* (day 1 vs. day 2 vs. day 3) was insignificant, all *p*s > .434.

4 Please note that the handling of outliers was described incorrectly in the preregistration of Study 2. Consistent with Study 1, outliers were handled as described in the Supporting Information.

5 Please note that the exclusion of participants responding “zero” minutes to procrastination failure T2 was not preregistered.

## References

[aphw12302-bib-0001] Adriaanse, M. A. , Oettingen, G. , Gollwitzer, P. M. , Hennes, E. P. , De Ridder, D. T. D. , & De Wit, J. B. F. (2010). When planning is not enough: Fighting unhealthy snacking habits by mental contrasting with implementation intentions (MCII). European Journal of Social Psychology, 40(7), 1277–1293. 10.1002/ejsp.730

[aphw12302-bib-0002] Adriaanse, M. A. , & Ten Broeke, P. (in preparation). Responding to self‐regulation failure: Understanding the setback effect of failure.

[aphw12302-bib-0003] Adriaanse, M. A. , Weijers, J. , De Ridder, D. T. D. , De Witt Huberts, J. , & Evers, C. (2014). Confabulating reasons for behaving bad: The psychological consequences of unconsciously activated behaviour that violates one's standards. European Journal of Social Psychology, 44(3), 255–266. 10.1002/ejsp.2005

[aphw12302-bib-0004] Ajzen, I. (2002). Perceived behavioral control, self‐efficacy, locus of control, and the theory of planned behavior. Journal of Applied Social Psychology, 32(4), 665–683. 10.1111/j.1559-1816.2002.tb00236.x

[aphw12302-bib-0005] Ashford, S. , Edmunds, J. , & French, D. P. (2010). What is the best way to change self‐efficacy to promote lifestyle and recreational physical activity? A systematic review with meta‐analysis. British Journal of Health Psychology, 15(Pt 2), 265–288. 10.1348/135910709X461752 19586583

[aphw12302-bib-0006] Bandura, A. (1978). Self‐efficacy: Toward a unifying theory of behavioral change. Advances in Behaviour Research and Therapy, 1(4), 139–161. 10.1016/0146-6402(78)90002-4

[aphw12302-bib-0007] Bar‐Anan, Y. , Wilson, T. D. , & Hassin, R. R. (2010). Inaccurate self‐knowledge formation as a result of automatic behavior. Journal of Experimental Social Psychology, 46(6), 884–894. 10.1016/j.jesp.2010.07.007

[aphw12302-bib-0008] Baron, R. M. , & Kenny, D. A. (1986). The moderator–mediator variable distinction in social psychological research: Conceptual, strategic, and statistical considerations. Journal of Personality and Social Psychology, 51, 1173–1182. 10.1037/0022-3514.51.6.1173 3806354

[aphw12302-bib-0009] Baumeister, R. F. , & Heatherton, T. F. (1996). Self‐regulation failure: An overview. Psychological Inquiry, 7(1), 1–15. 10.1207/s15327965pli0701_1

[aphw12302-bib-0010] Curry, S. , Marlatt, G. A. , & Gordon, J. R. (1987). Abstinence violation effect: Validation of an attributional construct with smoking cessation. Journal of Consulting and Clinical Psychology, 55(2), 145–149. 10.1037/0022-006X.55.2.145 3571666

[aphw12302-bib-0011] De Ridder, D. T. D. , Adriaanse, M. A. , & Fujita, K. (2018). The routledge international handbook of self‐ control in health and well‐being. New York, NY: Routledge. 10.4324/9781315648576

[aphw12302-bib-0012] Dolan, P. , & Galizzi, M. M. (2015). Like ripples on a pond: Behavioral spillovers and their implications for research and policy. Journal of Economic Psychology, 47, 1–16. 10.1016/j.joep.2014.12.00

[aphw12302-bib-0013] Harris, J. L. , Bargh, J. A. , & Brownell, K. D. (2009). Priming effects of television food advertising on eating behaviour. Health Psychology, 28, 404–413. 10.1037/a0014399 19594263 PMC2743554

[aphw12302-bib-0014] Heider, F. (1958). The psychology of interpersonal relations. New York: Wiley. 10.1037/10628-000

[aphw12302-bib-0015] Herman, C. P. , & Mack, D. (1975). Restrained and unrestrained eating. Journal of Personality, 43(4), 647–660. 10.1111/1467-6494.ep8970396 1206453

[aphw12302-bib-0016] Jansen, A. , Merckelbach, H. , Oosterlaan, J. , Tuiten, A. , & Van den Hout, M. (1988). Cognitions and self‐talk during food intake of restrained and unrestrained eaters. Behaviour Research and Therapy, 26(5), 393–398. 10.1016/0005-7967(88)90072-1 3190648

[aphw12302-bib-0017] Knäuper, B. , Roseman, M. , Johnson, P. J. , & Krantz, L. H. (2009). Using mental imagery to enhance the effectiveness of implementation intentions. Current Psychology, 28(3), 181–186. 10.1007/s12144-009-9055-0

[aphw12302-bib-0018] Luszczynska, A. , & Schwarzer, R. (2003). Planning and self‐efficacy in the adoption and maintenance of breast self‐examination: A longitudinal study on self‐regulatory conditions. Psychology and Health, 18(1), 93–108. 10.1080/0887044021000019358

[aphw12302-bib-0019] Maddux, J. E. , & Rogers, R. W. (1983). Protection motivation and self‐efficacy: A revised theory of fear appeals and attitude change. Journal of Experimental Social Psychology, 19(5), 469–479. 10.1016/0022-1031(83)90023-9

[aphw12302-bib-0020] Marlatt, G. A. , & George, W. H. (1984). Relapse prevention: Introduction and overview of the model. British Journal of Addiction, 79(3), 261–273. 10.1111/j.1360-0443.1984.tb00274.x 6595020

[aphw12302-bib-0035] Marlatt, G. A. , & Gordon, J. R. (1985). Relapse prevention, New York: Guilford Press.

[aphw12302-bib-0021] Marlatt, G. A. , & Witkiewtiz, K. (2005). Relapse prevention for alcohol and drug problems. In G. A. Marlatt & D. M. Donovan (Eds.), Relapse prevention: Maintenance strategies in the treatment of addictive behaviors (pp. 1–44). Guilford Press.

[aphw12302-bib-0022] Milyavskaya, M. , & Inzlicht, M. (2018). Attentional and motivational mechanisms of self‐control. In D. de Ridder , M. Adriaanse , & K. Fujita (Eds.), The Routledge international handbook of self‐control in health and well‐being (pp. 11–24). New York: Routledge.

[aphw12302-bib-0023] Nisbett, R. E. , & Wilson, T. D. (1977). Telling more than we can know: Verbal reports on mental processes. Psychological Review, 84, 231–259. 10.1037/0033-295X.84.3.231

[aphw12302-bib-0024] Oettingen, G. , Grant, H. , Smith, P. K. , Skinner, M. , & Gollwitzer, P. M. (2006). Unconscious goal pursuit: Acting in an explanatory vacuum. Journal of Experimental Social Psychology, 42, 668–675. 10.1016/j.jesp.2005.10.003

[aphw12302-bib-0025] Parks‐Stamm, E. J. , Oettingen, G. , & Gollwitzer, P. M. (2010). Making sense of one's actions in an explanatory vacuum: The interpretation of unconscious goal striving. Journal of Experimental Social Psychology, 46, 531–542. 10.1016/j.jesp.2010.02.004

[aphw12302-bib-0026] Schwarzer, R. (2008). Modeling health behavior change: How to predict and modify the adoption and maintenance of health behaviors. Applied Psychology. An International Review, 57, 1–29.

[aphw12302-bib-0027] Sheeran, P. , Gollwitzer, P. M. , & Bargh, J. A. (2013). Nonconscious processes and health. Health Psychology, 32, 460–473. 10.1037/a0029203 22888816

[aphw12302-bib-0028] Steinberg, D. M. , Tate, D. F. , Bennett, G. G. , Ennett, S. , Samuel‐Hodge, C. , & Ward, D. S. (2013). The efficacy of a daily self‐weighing weight loss intervention using smart scales and email. Obesity, 9, 1789–1797. 10.1002/oby.20396 PMC3788086 23512320

[aphw12302-bib-0029] Swoboda, C. M. , Miller, C. K. , & Wills, C. E. (2017). Impact of a goal setting and decision support telephone coaching intervention on diet, psychosocial, and decision outcomes among people with type 2 diabetes. Patient Education and Counseling, 7, 1367–1373. 10.1016/j.pec.2017.02.007 28215827

[aphw12302-bib-0030] Tanner, T. J. , Ferraro, R. , & Chartrand, T. L. (2008). Of chameleons and consumption: The impact of mimicry on choice and preferences. Journal of Consumer Research, 34, 754–766. 10.1086/522322

[aphw12302-bib-0031] Wagner, D. D. , & Heatherton, T. F. (2015). Self‐regulation and its failure: The seven deadly threats to self‐regulation. In M. Mikulincer & P. R. Shaver (Eds.), APA handbook of personality and social psychology: Vol 1. Attitudes and social cognition (p. Washington, D. C.). Washington, D. C: American Psychological Association.

[aphw12302-bib-0032] Weiss, E. C. , Galuska, D. A. , Khan, L. K. , & Serdula, M. K. (2006). Weight‐control practices among U.S. adults, 2001–2002. American Journal of Preventive Medicine, 31(1), 18–24. 10.1016/j.amepre.2006.03.016 16777538

[aphw12302-bib-0033] Wenzel, M. , Rowland, Z. , Hofmann, W. , & Kubiak, T. (2020). Setbacks in self‐control: Failing not mere resisting impairs subsequent self‐control. Social Psychological and Personality Science, 11(6), 782–790. 10.1177/1948550619888875

