# Supplementary material for: Beyond prevention: Regulating responses to self‐regulation failure to avoid a set‐back effect
Source: Appl Psychol Health Well Being. 2021 Aug 18;14(1):278–93. doi: 10.1111/aphw.12302 (PMC9291624; doi:10.1111/aphw.12302)
Supplement: Supplementary file 1 — Data S1. Supporting Information [file APHW-14-278-s001.docx]

Supplementary Information for

**Beyond Prevention: Regulating Responses to Self-Regulation Failure to Avoid a Set-Back Effect**

**This document includes:**

Supplementary Text 1: Study Materials

SI References

**Supplementary Text 1: Study Materials**

**Study 1**

**Measurement moment 1**

**Demographics**

First, we would like to ask you some basic questions about your demographics.

1. What is your age?
2. What is your gender?

male - female

1. What is the highest level of school you have completed or the highest degree you have received?

none - high/secondary school - associate degree - bachelor - master - PhD

1. Where do you live?

United States - United Kingdom – other

1. Is English your native language?

yes – no

1. For the current study, we are also interested in your BMI. Therefore, we want to ask you to indicate your height and your weight. First, please indicate in which unit of measurements you prefer to indicate your height and your weight.

Height:

- Feet and inches
- Centimetres

Weight:

- Pounds
- Kilograms

What is your height in **feet and inches**? [*text entry*]

*For example:*

*If your height is 5 feet 7 inches (5' 7"), you fill out*

● *Feet: 5*

● *Inches: 7*

What is your height in **centimetres?** [*text entry*]

What is your weight in **pounds** (lbs)? [*text entry*]

What is your weight in **kilograms**? [*text entry*]

1. To what extent do you currently in your everyday life try to limit your unhealthy food intake in any way?

[1 = Not at all, 7 = Very much]

***Restraint Scale (Herman & Polivy, 1980)***

With the next questionnaire we would like to measure how much you are occupied with your food intake. Please answer the questions as honestly as possible.

1. How often are you dieting?

never - rarely - sometimes - usually – always

1. What is the maximum amount of weight (in pounds) you have ever lost within one month?

0-4 - 5-9 - 10-14 - 15-19 - 20+

1. What is your maximum weight gain (in pounds) within a week?

0-1 - 1.1 - 2 - 2.1-3 - 3.1-5 - 5.1+

1. In a typical week, how much does your weight fluctuate?

0-1 - 1.1-2 - 2.1-3 - 3.1-5 - 5.1+

1. Would a weight fluctuation of 5 lb. affect the way you live your life?

not at all - slightly - moderately - very much

1. Do you eat sensibly in front of others and splurge alone?

never - rarely - sometimes - usually - always

1. Do you give too much time and thought to food?

never - rarely - sometimes - usually - always

1. Do you have feelings of guilt after overeating?

never - rarely - sometimes - usually - always

1. How conscious are you of what you're eating?

not at all - slightly - moderately - extremely

20. How many pounds over your desired weight were you at your maximum weight?

0-2 - 1-5 - 6-10 - 11-20 - 21

**Baseline eating questions**

We would now like to ask you some questions about your eating/dieting behaviour. Please indicate the extent to which the following statements apply to you:

***Intention to eat less unhealthily***

- - - 1. I feel motivated to refrain from unhealthy meals and snacks.

Totally disagree (1) – Totally agree (7)

- - - 1. I intend to refrain from unhealthy meals and snacks.

Totally disagree (1) – Totally agree (7)

- - - 1. I feel in control over refraining from unhealthy meals and snacks.

Totally disagree (1) – Totally agree (7)

- - - 1. I feel confident in my abilities to refrain from unhealthy meals and snacks.

Totally disagree (1) – Totally agree (7)

- - - 1. I feel that it is worthwhile to refrain from unhealthy meals and snacks for the rest of the day.

Totally disagree (1) – Totally agree (7)

- - - 1. I feel that it is worthwhile to refrain from unhealthy meals and snacks from now on.

Totally disagree (1) – Totally agree (7)

***Failure/ Success on a typical weekend day.***

***Friday.***

Please take a moment to imagine **your** **typical Friday**. Try to create a mental image of a typical Friday morning. And now, do the same for the afternoon and evening on a typical Friday. Imagine this as vividly as possible.

After creating this mental image of a typical Friday, please answer the following questions:

1. On a typical Friday, to what extent do you feel like you successfully follow your diet?

*Slider from* unsuccessful 0 – 100 % successful

2. On a typical Friday, how many times do you eat something that is **not** in line with your dieting goal?

*0 - 20+ [dropdown]*

***Saturday.***

Please take a moment to imagine **your** **typical Saturday**. Try to create a mental image of a typical Saturday morning. And now, do the same for the afternoon and evening on a typical Saturday. Imagine this as vividly as possible.

After creating this mental image of a typical Saturday, please answer the following questions:

1. On a typical Saturday, to what extent do you feel like you successfully follow your diet?

*Slider from* unsuccessful 0 – 100 % successful

2. On a typical Saturday, how many times do you eat something that is **not** in line with your dieting goal?

0 - 20+ *[dropdown]*

***Sunday.***

Please take a moment to imagine **your** **typical Sunday**. Try to create a mental image of a typical Sunday morning. And now, do the same for the afternoon and evening on a typical Sunday. Imagine this as vividly as possible.

After creating this mental image of a typical Sunday, please answer the following questions:

1. On a typical Sunday, to what extent do you feel like you successfully follow your diet?

*Slider from* unsuccessful 0 – 100 % successful

2. On a typical Sunday, how many times do you eat something that is **not** in line with your dieting goal?

0 - 20+ *[dropdown]*

**Lay beliefs in the snowball effect and the what the hell effect**

Please indicate to what extent you agree with the following statements regarding your dieting behaviour.

1. Once I eat something that is not in line with my dieting goal, I feel that I am more likely to do it again.

[Strongly disagree      1          2          3          4          5          6          7          Strongly agree]

2. When I eat something that is not in line with my dieting goal, I feel there is no point in adhering to my diet for the rest of the day, so I abandon my dieting standards for that day.

[Strongly disagree      1          2          3          4          5          6          7          Strongly agree]

**Baseline procrastination questions**

We would now like to ask you some questions about your tendencies to procrastinate. In the following questions, we refer to procrastination in general, so procrastinating on any tasks or chore or activity, such as studying for an exam, doing the dishes, making a phone call etc. Please indicate the extent to which the following statements apply to you:

***Intention to Procrastinate.***

1. I feel motivated to refrain from procrastination.

Totally disagree (1) – Totally agree (7)

1. I intend to refrain from procrastination.

Totally disagree (1) – Totally agree (7)

1. I feel in control over refraining from procrastination.

Totally disagree (1) – Totally agree (7)

1. I feel confident in my abilities to refrain from procrastination.

Totally disagree (1) – Totally agree (7)

1. I feel that it is worthwhile to refrain from procrastination for the rest of the day.

Totally disagree (1) – Totally agree (7)

1. I feel that it is worthwhile to refrain from procrastination from now on.

Totally disagree (1) – Totally agree (7)

***Procrastination on Typical Weekend Days.***

***Friday.***

Please take a moment to imagine **your** **typical Friday**. Try to create a mental image of a typical Friday morning. And now, do the same for the afternoon and evening on a typical Friday. Imagine this as vividly as possible.

After creating this mental image of a typical Friday, please answer the following question:

1. On a typical Friday, to what extent do you feel like you successfully refrain from procrastinating on tasks?

*Slider from* unsuccessful 0 – 100 % successful

2. Over the course of a typical Friday, how many minutes do you spend procrastinating on tasks? *(Only use digits please)*

*[text entry]*

***Saturday.***

Please take a moment to imagine **your** **typical Saturday**. Try to create a mental image of a typical Saturday morning. And now, do the same for the afternoon and evening on a typical Saturday. Imagine this as vividly as possible.

After creating this mental image of a typical Saturday, please answer the following question:

1. On a typical Saturday, to what extent do you feel like you successfully refrain from procrastinating on tasks?

*Slider from* unsuccessful 0 – 100 % successful

2. Over the course of a typical Saturday, how many minutes do you spend procrastinating on tasks? *(Only use digits please)*

*[text entry]*

***Sunday.***

Please take a moment to imagine **your** **typical Sunday**. Try to create a mental image of a typical Sunday morning. And now, do the same for the afternoon and evening on a typical Sunday. Imagine this as vividly as possible.

After creating this mental image of a typical Sunday, please answer the following question:

1. On a typical Sunday, to what extent do you feel like you successfully refrain from procrastinating on tasks?

*Slider from* unsuccessful 0 – 100 % successful

2. Over the course of a typical Sunday, how many minutes do you spend procrastinating on tasks? *(Only use digits please)*

*[text entry]*

**Interventions (eating + procrastination)**

On the following screen, you will be presented with scientific findings about successful goal pursuit. In particular, you will be introduced to a certain **mindset shift** that might help you in the future to better adhere to your long-term goal after you failed sticking to it.

Please read the following text carefully. It is very important that you pay close attention, since we will later invite you to use the described **mindset shift** yourself.

***Eating***

*Informative text*

Mindset shift for successful goal pursuit

When you violated your diet, do you ever wonder how to get back on track?

Research has shown that whether you can get back on track actually has a lot to do with the way people think about the *causes* of their unhealthy eating behaviour.

A helpful way for you to think about why you failed your diet is to focus on the factors *outside of you,* such as the environment, or the people around you that influenced your behaviour. These factors have a tremendous, but often underestimated, effect on your eating behaviour.

Research has shown that acknowledging these kinds of external factors when failing your diet may be a helpful way to get you back on track.

*Intervention text*

As was explained before, we would now like you to **apply** **the** **mindset shift** you just read about to your own behaviour in the upcoming days. It might help you to stick to your dieting goal in the upcoming days.

Again, it is really important that you pay close attention to the following instructions. To help you apply the **mindset shift** you just learned about, we would like you to make the following plan:

*Implementation intention*

“If I fail to adhere to my dieting goal, then …

… I will reflect on the external factors that contributed to this failure,

and will continue to pursue my dieting goal as usual.”

Please commit to this plan and take two minutes to repeat it in your mind a few times. *(insert plan here)*

This is important because we would like you to try and stick to your plan in the upcoming 3 days.

Research has shown that this type of planning works best when the specific plan is pictured in your mind.

Therefore, please take the next two minutes to try to envision yourself enacting your plan: *(insert implementation intention per condition)*. Imagine that you are in this situation and that you are enacting this plan. Try to imagine enacting your plan as vividly as possible.

Now please type the plan you just envisioned yourself below.

As was explained before, we would like you to apply this **mindset shift** you just learned in the upcoming 3 days. So, every time you fail to adhere to your dieting goal, please try to enact the plan you just practiced and envisioned.

***Procrastination***

*Informative text*

Mindset shift for successful goal pursuit

When you procrastinate, do you ever wonder how to get back on track?

Research has shown that whether you get back on track actually has a lot to do with the way people think about the *causes* of their procrastination.

A helpful way for you to think about why you procrastinated is to focus on the factors *outside of you,* such as the environment, or the people around you that influenced your behaviour. These factors have a tremendous, but often underestimated, effect on your behaviour.

Research has shown that acknowledging these kinds of external factors when procrastinating may be a helpful way to get you back on track.

*Intervention text*

As was explained before, we would now like you to **apply the mindset shift** you just read about to your own behaviour in the upcoming days. It might aid your goal pursuit in the upcoming days.

Again, it is really important that you pay close attention to the following instructions. To help you apply the **mindset shift** you just learned about, we would like you to make the following plan:

*Implementation intention*

“If I fail to pursue my goal due to procrastination, then …

...I will reflect on the external factors that contributed to my procrastinating behaviour,

and will continue to pursue my goal as usual.”

Please commit to this plan and take two minutes to repeat it in your mind a few times. *(insert plan here)*

This is important because we would like you to try and stick to your plan in the upcoming 3 days.

Research has shown that this type of planning works best when the specific plan is pictured in your mind.

Therefore, please take the next two minutes to try to envision yourself enacting your plan: *(insert implementation intention per condition)*. Imagine that you are in this situation and that you are enacting this plan. Try to imagine enacting your plan as vividly as possible.

Now please type the plan you just envisioned yourself below.

As was explained before, we would like you to apply this **mindset shift** you just learned in the upcoming 3 days. So, every time you fail to pursue your goal, please try to enact the plan you just practiced and envisioned.

## Measurement moment 2 (Monday)

**Eating Behavior**

In the first part of this study, you indicated that you restrict your food intake with the goal to manage your weight. The following questions are about **your dieting behaviour** in the past three days **related to this specific goal**. So please answer the following questions while remembering your dieting behaviour during each of these three days. We will ask you to recall your behaviour for each day separately.

In order to help you to recall your behaviour for the past three days, we provide you with the image of a calendar

*[image calendar]*

Please indicate today’s day and date ….., and make sure you find it on the calendar.

***Day 3 (Sunday)***

[*image calendar*]

Please indicate **yesterday’s** day and date …

Take a moment to think back to **yesterday.**

1. To what extent do you feel like you successfully followed your diet during this day?

*Slider from* unsuccessful 0 – 100 % successful

2. How many times during this day did you eat something that was **not** in line with your dieting goal?

0 - 20+ *[dropdown]*

3. To what extent do you feel like you were able to correctly recall your dieting behavior during this day in order to answer the above questions?

Not at all 1 2 3 4 5 6 7 Very much

***Day 2 (Saturday)***

[*image calendar*]

Please indicate the day and date of **two days ago** …

Take a moment to think back to **two days ago.**

1. To what extent do you feel like you successfully followed your diet during this day?

*Slider from* unsuccessful 0 – 100 % successful

2. How many times during this day did you eat something that was **not** in line with your dieting goal?

0 - 20+ *[dropdown]*

3. To what extent do you feel like you were able to correctly recall your dieting behavior during this day in order to answer the above questions?

Not at all 1 2 3 4 5 6 7 Very much

***Day 1 (Friday)***

[*image calendar*]

Please indicate the day and date of **three days ago** …

Take a moment to think back to **three days ago.**

1. To what extent do you feel like you successfully followed your diet during this day?

*Slider from* unsuccessful 0 – 100 % successful

2. How many times during this day did you eat something that was **not** in line with your dieting goal?

0 - 20+ *[drop down]*

3. To what extent do you feel like you were able to correctly recall your dieting behavior during this day in order to answer the above questions?

Not at all 1 2 3 4 5 6 7 Very much

**Procrastination Behaviour**

The following questions are about **your procrastination on tasks or chores or activities** in the past three days. So please answer the following questions while remembering your procrastination behaviour during each of these three days. We will ask you to recall your behaviour for each day separately.

In order to help you to recall your behaviour for the past three days, we provide you with the image of a calendar

*[image calendar]*

Please indicate today’s day and date ….., and make sure you find it on the calendar.

***Day 3 (Sunday)***

[*image calendar*]

Please indicate **yesterday’s** day and date …

Take a moment to think back to **yesterday.**

1. During this day, to what extent do you feel like you successfully refrained from procrastinating on tasks?

*Slider from* unsuccessful 0 – 100 % successful

2. Over the course of the day, how many minutes did you spend procrastinating on tasks?

*[text entry]*

3. To what extent do you feel like you were able to correctly recall your procrastination behaviour during this day in order to answer the above questions?

Not at all 1 2 3 4 5 6 7 Very much

***Day 2 (Saturday)***

[*image calendar*]

Please indicate the day and date of **two days ago** …

Take a moment to think back to **two days ago.**

1. During this day, to what extent do you feel like you successfully refrained from procrastinating on tasks?

*Slider from* unsuccessful 0 – 100 % successful

2. Over the course of the day, how many minutes did you spend procrastinating on tasks?

*[text entry]*

3. To what extent do you feel like you were able to correctly recall your procrastination behaviour during this day in order to answer the above questions?

Not at all 1 2 3 4 5 6 7 Very much

***Day 1 (Friday)***

[*image calendar*]

Please indicate the day and date of **three days ago** …

Take a moment to think back to **three days ago.**

1. During this day, to what extent do you feel like you successfully refrained from procrastinating on tasks?

*Slider from* unsuccessful 0 – 100 % successful

2. Over the course of the day, how many minutes did you spend procrastinating on tasks?

*[text entry]*

3. To what extent do you feel like you were able to correctly recall your procrastination behaviour during this day in order to answer the above questions?

Not at all 1 2 3 4 5 6 7 Very much

**Control Questions**

Please think back to the first part of this study. After answering questions about yourself and your dieting behavior, did you read a short text explaining a **mindset shift** you can use to successfully pursue your goal?

Yes or No

*(Only if yes on previous question)*

Which **mindset shift** were you asked to apply? In other words, every time you failed to adhere to your dieting goal / your goal to accomplish tasks, what were you instructed to do?

*[text entry]*

In the past 3 days, to what extent do you feel like you were able to implement the **mindset shift** you learned in the first part of this study?

Not at all 1 2 3 4 5 6 7 Very much

In the past 3 days, how helpful do you think that **mindset shift** was to reach your dieting goal?

Not at all 1 2 3 4 5 6 7 Very much

*(everyone again)*

In the past 3 days,whenever you ate something that was not in line with your dieting goal, to what extent did you reflect on the external factors that contributed to that failure?

Not at all 1 2 3 4 5 6 7 Very much

**Study 2**

**Measurement moment 1**

***Demographics***

First, we would like to ask you some basic questions about your demographics.

1. What is your age?
2. What is your gender?

male – female – other

1. What is the highest level of school you have completed or the highest degree you have received?

none - high/secondary school - associate degree - bachelor - master - PhD

1. Where do you live?

United States - United Kingdom – other

1. Is English your native language?

yes – no

***Baseline procrastination questions***

At this moment, the whole world is experiencing the consequences of the Corona virus outbreak. Next to financial and social difficulties, most people are forced to work and study from home, which also has its challenges.

For this study, we are interested in procrastination behaviour specifically.

In the past month, have you procrastinated on any tasks, chore or activity?

Yes – No

Are you currently trying to actively minimize procrastination behaviour in any way?

[Not at all 1          2          3          4          5          6          7          Very much]

***General procrastination scale***

People may use the following statements to describe themselves. For each statement, decide whether the statement is uncharacteristic or characteristic of you using the following 5 point scale. Note that the 3 on the scale is Neutral – the statement is neither characteristic nor uncharacteristic of you.

1. I often find myself performing tasks that I had intended to do days before

[Extremely uncharacteristic of me 1 2 3 4 5 Extremely characteristics of me]

1. I often miss concerts, sporting events, or other events alike because I don’t around to buying tickets on time.

[Extremely uncharacteristic of me 1 2 3 4 5 Extremely characteristics of me]

1. When planning a party, I make the necessary arrangements well in advance.

[Extremely uncharacteristic of me 1 2 3 4 5 Extremely characteristics of me]

1. When it is time to get up in the morning, I most often get right out of bed.

[Extremely uncharacteristic of me 1 2 3 4 5 Extremely characteristics of me]

1. An e-mail may sit for hours after I write it before sending it.

[Extremely uncharacteristic of me 1 2 3 4 5 Extremely characteristics of me]

1. I generally return phone calls promptly.

[Extremely uncharacteristic of me 1 2 3 4 5 Extremely characteristics of me]

1. Even with jobs that require little else except sitting down and doing them, I find they seldom get done for days.

[Extremely uncharacteristic of me 1 2 3 4 5 Extremely characteristics of me]

1. I usually make decisions as soon as possible/

[Extremely uncharacteristic of me 1 2 3 4 5 Extremely characteristics of me]

1. I generally delay before staring on work I have to do.

[Extremely uncharacteristic of me 1 2 3 4 5 Extremely characteristics of me]

1. When traveling, I usually have to rush in preparing to arrive at the airport or station at the appropriate time.

[Extremely uncharacteristic of me 1 2 3 4 5 Extremely characteristics of me]

1. When preparing to get out, I am seldom caught having to do something at the last minute.

[Extremely uncharacteristic of me 1 2 3 4 5 Extremely characteristics of me]

1. In preparing for some deadline, I often waste time be doing other things.

[Extremely uncharacteristic of me 1 2 3 4 5 Extremely characteristics of me]

***Baseline self-efficacy and intention***

**We would now like to ask you some questions about your tendencies to procrastinate**. In the following questions, we refer to procrastination in general, so procrastinating on any tasks or chore or activity, such as studying for an exam, doing the dishes, making a phone call etc. Please indicate the extent to which the following statements apply to you:

I intend to minimize my procrastination behaviour.

Totally disagree (1) – Totally agree (7)

I plan to minimize my procrastination behaviour.

Totally disagree (1) – Totally agree (7)

I feel in control over minimizing my procrastination behaviour.

Totally disagree (1) – Totally agree (7)

I feel confident in my abilities to minimize my procrastination behaviour.

Totally disagree (1) – Totally agree (7)

***Baseline procrastination.***

Now, we would like to ask you a few questions about your procrastination behaviour during a typical week.

***Monday***

Please take a moment to imagine **a typical Monday**. Try to create a mental image of a typical Monday morning. And now, do the same for the afternoon and evening on a typical Monday. Imagine this as vividly as possible.

After creating this mental image of a typical Monday, please answer the following question:

Over the course of a typical Monday, how many minutes do you *approximately* spend procrastinating on tasks? (*Only use digits please*)

*[text entry]*

***Tuesday***

Please take a moment to imagine **a typical Tuesday**. Try to create a mental image of a typical Tuesday morning. And now, do the same for the afternoon and evening on a typical Tuesday. Imagine this as vividly as possible.

After creating this mental image of a typical Tuesday, please answer the following question:

Over the course of a typical Tuesday, how many minutes do you *approximately* spend procrastinating on tasks? (*Only use digits please*)

*[text entry]*

***Wednesday.***

Please take a moment to imagine **a typical Wednesday**. Try to create a mental image of a typical Wednesday morning. And now, do the same for the afternoon and evening on a typical Wednesday. Imagine this as vividly as possible.

After creating this mental image of a typical Wednesday, please answer the following question:

Over the course of a typical Wednesday, how many minutes do you *approximately* spend procrastinating on tasks? (*Only use digits please*)

*[text entry]*

***Intervention***

*Introduction*

**Dealing with procrastination**

On the following screen, you will be presented with scientific findings about how to successfully deal with procrastination. In particular, you will be introduced to a certain **mindset shift** that might help you in the future to better adhere to your goal to minimize procrastination after you failed sticking to it.

Please read the following text carefully. It is very important that you pay close attention, since we will later invite you to use the described **mindset shift** yourself.

*Text*

**Mindset shift for successful goal pursuit**

When you procrastinate, do you ever wonder how to get back on track?

Research has shown that whether you get back on track actually has a lot to do with the way people think about the *causes* of their procrastination.

A helpful way for you to think about why you procrastinated is to focus on the factors *outside of you*, such as distractions in your direct environment, or the people around you that influenced your behaviour. These factors have a tremendous, but often underestimated, effect on your behaviour.

Research has shown that acknowledging these kinds of external factors when procrastinating may be a helpful way to get you back on track.

*Implementation intention*

As was explained before, we would now like you to **apply the mindset shift** you just read about to your own behaviour in the upcoming days. It might aid your goal pursuit in the upcoming days.

Again, it is really important that you pay close attention to the following instructions. To help you apply the **mindset shift** you just learned about, we would like you to make the following plan:

*“If I have procrastinated, then I will reflect on the external factors that contributed to my procrastinating behaviour, and will continue to pursue my goal as usual.”*

Please commit to this plan and take two minutes to repeat it in your mind a few times.

Please commit to this plan and take two minutes to repeat it in your mind a few times.

If I have procrastinated, then I will reflect on the external factors that contributed to my procrastinating behaviour, and will continue to pursue my goal as usual.”

This is important because we would like you to try and stick to your plan in the upcoming 3 days.

Research has shown that this type of planning works best when the specific plan is pictured in your mind.

Therefore, please take the next two minutes to try to envision yourself enacting your plan:

“If I have procrastinated, then I will reflect on the external factors that contributed to my procrastinating behaviour, and will continue to pursue my goal as usual.”

Imagine that you are in this situation and that you are enacting this plan. Try to imagine enacting your plan as vividly as possible.

Now please type the plan you just envisioned yourself below.

*[Text entry]*

As was explained before, we would like you to apply this **mindset shift** you just learned in the upcoming 3 days. So, whenever you have failed to minimize your procrastination behaviour as intended, please try to enact the plan you just practiced and envisioned.

## Measurement moment 2

***Self-efficacy and intention T1***

In comparison to before I completed this study, I intend to minimize my procrastination behaviour.

Totally disagree (1) – Totally agree (7)

In comparison to before I completed this study, I plan to minimize my procrastination behaviour.

Totally disagree (1) – Totally agree (7)

In comparison to before I completed this study, I feel in control over minimizing my procrastination behaviour.

Totally disagree (1) – Totally agree (7)

In comparison to before I completed this study, I feel confident in my abilities to minimize my procrastination behaviour.

Totally disagree (1) – Totally agree (7)

***Procrastination Behaviour T2***

The following questions are about **your procrastination on tasks or chores or activities** over the past three days. So, please answer the following questions while remembering your procrastination behaviour during each of these three days. We will ask you to recall your behaviour for each day separately.

In order to help you to recall your behaviour for the past three days, we provide you with the image of a calendar.

*[image calendar]*

Please indicate today’s day and date ….., and make sure you find it on the calendar.

***Day 3***

[*image calendar*]

Please indicate **yesterday’s** day and date …

Take a moment to think back to **yesterday.**

Over the course of the day, how many minutes did you spend procrastinating on tasks? *(only use digits please)*

*[text entry]*

To what extent do you feel like you were able to correctly recall your procrastination behaviour during this day in order to answer the above questions?

Not at all 1 2 3 4 5 6 7 Very much

***Day 2***

[*image calendar*]

Please indicate the day and date of **two days ago** …

Take a moment to think back to **two days ago.**

Over the course of the day, how many minutes did you spend procrastinating on tasks?

*[text entry]*

To what extent do you feel like you were able to correctly recall your procrastination behaviour during this day in order to answer the above questions?

Not at all 1 2 3 4 5 6 7 Very much

***Day 1***

[*image calendar*]

Please indicate the day and date of **three days ago** …

Take a moment to think back to **three days ago.**

Over the course of the day, how many minutes did you spend procrastinating on tasks?

*[text entry]*

To what extent do you feel like you were able to correctly recall your procrastination behaviour during this day in order to answer the above questions?

Not at all 1 2 3 4 5 6 7 Very much

***Manipulation check***

Please think back to the first part of this study. After answering questions about yourself and your dieting behavior, did you read a short text explaining a **mindset shift** you can use to successfully pursue your goal?

Yes or No

Which **mindset shift** were you asked to apply? In other words, every time you failed to accomplish tasks due to procrastination, what were you instructed to do?

*[text entry]*

In the past 3 days, to what extent do you feel like you were able to implement the **mindset shift** you learned in the first part of this study?

Not at all 1 2 3 4 5 6 7 Very much

In the past 3 days, how helpful do you think that **mindset shift** was to minimize procrastination behaviour?

Not at all 1 2 3 4 5 6 7 Very much

***Internal / external attribution***

In the past 3 days,whenever you procrastinated, to what extent did you reflect on the **external** factors that contributed to that failure?

Not at all 1 2 3 4 5 6 7 Very much

In the past 3 days,whenever you procrastinated, to what extent did you reflect on the **internal** factors that contributed to that failure?

Not at all 1 2 3 4 5 6 7 Very much

**References**

Cialdini, R. B., Trost, M. R., & Newsom, J. T. (1995). Preference for consistency:

The development of a valid measure and the discovery of surprising behavioral

implications. Journal of Personality and Social Psychology,69, 318–328.

Herman, C. P., & Polivy, J. (1980). Restrained eating. In A. J. Stunkard, *Obesity* (pp. 108–225). Saunders.
